# From the Ground to the Clinic: The Evolution and Adaptation of Fungi

**DOI:** 10.3390/jof12010008

**Published:** 2025-12-23

**Authors:** Dario Corrêa-Junior, Daniel Zamith-Miranda, Susana Frases, Joshua D. Nosanchuk

**Affiliations:** 1 Laboratório de Biofísica de Fungos, Instituto de Biofísica Carlos Chagas Filho, Universidade Federal do Rio de Janeiro, Rio de Janeiro 21941-902, Brazil; dariojunior@biof.ufrj.br (D.C.-J.); susanafrases@biof.ufrj.br (S.F.); 2 Departments of Medicine (Division of Infectious Diseases) and Microbiology and Immunology, Albert Einstein College of Medicine, Bronx, NY 10461, USA; daniel.zamithmiranda@einsteinmed.edu; 3 Rede Micologia, Fundação de Amparo à Pesquisa do Estado do Rio de Janeiro—FAPERJ, Rio de Janeiro 21941-902, Brazil

**Keywords:** fungal pathogenesis, hydrolytic enzymes, thermotolerance, melanin, antifungal resistance, biofilms, fungal diagnostics, One Health, fungal adaptation, emerging therapies

## Abstract

Fungi constitute a diverse kingdom of eukaryotic organisms with remarkable adaptability, ranging from saprophytic decomposers to lethal human pathogens. This review synthesizes current insights into fungal adaptations that underline pathogenesis, focusing on enzymatic strategies including hydrolytic enzymes, metabolic and physiological plasticity such as thermotolerance and nutrient flexibility, and evasion of host immunity via mechanisms like melanin production and biofilm formation. We detail fungal survival tactics including spore formation and genomic and epigenetic plasticity, which contribute to resilience and evolution under environmental and host-imposed stresses. The escalating emergence of antifungal resistance and the global impact of environmental changes underscore urgent clinical challenges. Advances in diagnostics, novel therapeutics incorporating AI-assisted drug discovery, and integrated One Health approaches are poised to combat this growing threat. This comprehensive overview aims to guide future research and inform clinical management of fungal infections in an era of dynamic microbial evolution and environmental upheaval.

## 1. Introduction: Why Study Fungal Adaptations in Pathogenic Fungi

The fungal kingdom comprises eukaryotic, heterotrophic organisms with chitinous cell walls and diverse morphologies, ranging from unicellular yeasts to filamentous fungi and dimorphic species capable of switching forms depending on environmental and/or host conditions [1]. Estimates suggest 2.2 to 3.8 million fungal species exist, but only about 120,000 have been formally described [2]. Among these, some are pathogenic to humans and animals, underscoring the medical relevance of fungal adaptation.

Human pathogenic fungi exploit diverse ecological niches, from soil and water to host-associated environments, showing remarkable physiological and metabolic plasticity. They have evolved strategies to survive hostile conditions, evade host immune responses, produce bioactive secondary metabolites, and transition between saprophytic and parasitic lifestyles [3]. These adaptations underpin their ability to cause infections ranging from superficial cutaneous mycoses to invasive systemic diseases [4].

The capacity to transition from environmental reservoirs to human or animal hosts involves complex evolutionary adaptations enabling survival under stresses such as temperature, oxidative stress, and immune defenses [5]. Genetic variation and regulatory flexibility facilitate antifungal resistance and emergence of novel pathogenic strains [5]. Metabolic and morphological plasticity, including switching between yeast and filamentous forms, support survival in host tissues and persistence under antifungal treatment [6]. This review focuses on adaptation mechanisms in pathogenic fungal species, emphasizing enzymatic strategies, temperature tolerance, and physiological adaptations facilitating host colonization, integrating ecological, molecular, and genetic perspectives.

## 2. Hydrolytic Enzymes

Fungi play a vital role in decomposing terrestrial organic matter such as lignocellulose, significantly contributing to nutrient cycling [7]. Their capacity to secrete diverse extracellular enzymes allows for the breakdown of recalcitrant compounds unusable by other organisms [8]. Mycorrhizal fungi mobilize nitrogen and phosphorus from organic sources, influencing nutrient cycles [9]. Decomposer succession with varied catabolic capabilities promotes fungal diversity over time, with soil fauna also impacting decomposer community functioning [7]. Biologically active metabolites suppress competitors and/or predators, shaping community dynamics during organic matter decomposition. Fungal enzymatic diversity underlies ecological success across habitats, including extreme environments, by facilitating nutrient mobilization, substrate degradation, and interactions with other organisms. For instance, hyphal growth and resource partitioning contribute to ecosystem stability and fungal persistence [10].

Pathogenic fungi use hydrolytic enzymes to degrade host tissues nutrient acquisition as well as for facilitating colonization and invasion. Many of these enzymes are homologous to those involved in environmental decomposition but are co-opted to breach human host barriers. Carbohydrate-active enzymes such as cellulases, hemicellulases, pectinases, chitinases, glucanases, and proteases are key players [11]. For example, phytopathogenic fungi use cellulases and pectinases to penetrate cell walls, whereas entomopathogens secrete chitinases and proteases to degrade insect exoskeletons [8], and human pathogens produce glucanases and proteases for tissue invasion and immune evasion [12]. Fungi can utilize a broad spectrum of carbon sources, including simple sugars (glucose, fructose, galactose), amino acids, organic acids, and host-derived macromolecules such as lipids and polysaccharides [13,14,15]. The production of these enzymes is finely regulated by global gene expression in response to host-derived substrates and environmental factors, adjusting fungal metabolism secreting effectors, enzymes, and secondary metabolites to withstand immunity and environmental stress [16].

A key feature exhibited by fungi that survive in different environments is their remarkable metabolic flexibility that allows them to adapt to the variable and often nutritionally limited conditions within host environments. For example, *Candida albicans*, a species that has adapted to become a human commensal, exhibits versatile carbon metabolism enabling colonization of mucosal surfaces, bloodstream, and internal tissues by metabolizing diverse sugars and amino acids. In contrast, *Nakaseomyces glabrata* (formerly Candida glabrata), another human commensal, shows a narrower substrate range due to loss of certain metabolic pathways, such as the ability to catabolize galactose or allantoin, reflecting evolutionary adaptations to its specialized host niches. *Aspergillus fumigatus*, an environmental saprobe and opportunistic pathogen, retains a broad capacity to exploit various fermentable and non-fermentable carbon compounds, which aids survival in both environmental reservoirs and human hosts [17].

Nitrogen metabolism in fungal pathogens is equally versatile. Fungi can assimilate preferred nitrogen sources like ammonium and glutamine but are also capable of utilizing urea, peptides, amino acids, polyamines, and other nitrogen-containing compounds. This is critical within host tissues where nitrogen sources are often limited by nutritional immunity. For instance, *Cryptococcus neoformans* modulates capsule synthesis, a key virulence factor, in response to nitrogen availability, demonstrating a direct link between nitrogen metabolism and pathogenicity [18,19,20]. Additionally, fungal pathogens employ protein recycling and autophagy pathways to overcome nitrogen starvation during infection. *C. albicans* upregulates vacuolar proteases and ubiquitin-dependent degradation pathways to recycle intracellular proteins under nutrient-limited conditions, supporting survival within macrophages and contributing to virulence [21]. Similarly, pathogens such as *N. glabrata* and *C. neoformans* rely on autophagy for full virulence, reflecting their preference for intracellular lifestyle adaptations [22]. The broad metabolic diversity and resource acquisition strategies of various species are further summarized (see Table 1 for detailed examples).

The role of degradative enzymes as virulence factors is a central concept in fungal pathogenesis, largely established through foundational studies in *Candida albicans*. In this archetypal pathogen, secreted aspartic proteinases (Saps) are key virulence factors, facilitating tissue invasion by degrading host cell membranes, surface glycoproteins, and extracellular matrix components. Therefore, the presence of similar proteinase activity in environmental or emerging fungi, such as those isolated in this study, is considered a strong indicator of potential virulence [36,37].

The *C. albicans* genome contains a multigene family encoding at least 10 SAP proteins, which are differentially expressed during distinct infection stages, enabling precise regulation of proteolytic activity to suit environmental and host cues [38]. Notably, genes such as *SAP4*, *SAP5*, and *SAP6* are predominantly upregulated during tissue invasion and systemic infection, highlighting their role in pathogenic progression [39]. Besides *C. albicans*, other fungal pathogens also rely on extracellular enzymes for host tissue penetration. Dermatophytes secrete keratinases, specialized proteases that degrade keratin in skin and nails, facilitating superficial mycoses [40]. *A. fumigatus* produces elastases that are critical for pulmonary tissue invasion and dissemination, especially in immunocompromised patients [41]. Additionally, phospholipases secreted by *Candida* and *Cryptococcus* species contribute to host membrane disruption and immune evasion, underscoring their multifaceted role in fungal pathogenesis [40,41,42].

The functional importance of these enzymes in infection is further supported by gene disruption studies, wherein mutants deficient in key hydrolytic enzymes exhibit attenuated virulence, reduced tissue invasion, and impaired biofilm formation. These findings not only clarify enzyme roles in fungal pathogenicity but also highlight promising therapeutic targets for antifungal drug development [42]. The dual roles of these enzymes in decomposition, invasion, and immune evasion are exemplified across major pathogenic species (Table 2).

Fungal biofilms are structured communities embedded in extracellular matrix and adhere to biotic and abiotic surfaces, including medical devices like catheters and prosthetics [48,49]. Biofilms confer survival advantages including resistance to antifungal agents and host immune defenses [50]. Fungi such as *Candida* spp., *Aspergillus fumigatus*, and *C. neoformans* form structured biofilms involving extracellular matrix production and secretion of hydrolytic enzymes, aiding adhesion, invasion, and persistence [51,52]. During biofilm development, *Candida* species, filamentous fungi such as *Aspergillus*, and encapsulated yeasts like *Cryptococcus* produce a variety of extracellular hydrolytic enzymes, including proteases, phospholipases, and lipases, that facilitate key processes such as adhesion to surfaces, tissue invasion, and nutrient acquisition [53]. This coordinated expression is tightly linked with fungal morphogenetic transitions (e.g., yeast-to-hyphae switch in *C. albicans*) and stress response pathways, enhancing the fungi’s ability to survive and thrive in hostile host microenvironments [51]. Additionally, hydrolytic enzymes serve in biofilm maturation by modulating the extracellular matrix composition, facilitating dispersal of fungal cells. Enzymatic degradation of host tissues also allows invasion beyond the biofilm’s immediate niche, contributing to systemic dissemination [54].

*C. neoformans* secretes enzymes that modulate its polysaccharide capsule structure and mask pathogen-associated molecular patterns (PAMPs), dampening recognition by host immune cells and promoting immune evasion, by reduced phagocytosis and impairment of inflammatory responses [55]. Alternatively, *A. fumigatus* produces an array of proteases, including elastases and metalloproteases, that degrade pulmonary tissue not only promoting tissue invasion but also fostering immunosuppression [43]. Recent studies also revealed cell wall-associated glucanases and other hydrolases from entomo- and phytopathogenic fungi that remodel surface PAMPs, which reduces immune activation by avoiding detection, interfering with immune signaling, and protecting against oxidative bursts by immune cells [56].

The role of hydrolytic enzymes and metabolic flexibility in fungal virulence is well-established, yet clinical progress is hindered by knowledge gaps in enzymatic regulation, particularly the “master switches” that control the transition between saprophytic metabolism and tissue aggression in opportunistic pathogens. There is a controversy regarding the actual quantitative contribution of specific enzymes in vivo, as functional redundancy and high enzymatic plasticity suggest that the loss of one pathway can be compensated by others, necessitating continuous reassessment of virulence profiles, such as in the case of the reclassification of *Candidozyma auris*. Future directions must employ omics and single-cell approaches to map the enzymatic and metabolic dynamics within host microenvironments, aiming to identify inhibitors that can selectively modulate enzyme families or disrupt metabolic switches crucial for host survival, paving the way for the development of next-generation antifungals.

## 3. Thermotolerance

Fungi exhibit remarkable resilience in extreme habitats inhospitable to most life forms [57]. Extremotolerant species, such as black fungi exemplified by *Exophiala dermatitidis*, thrive in deserts, high UV exposure zones, and oligotrophic niches, aided by protective melanin pigments, mycosporines, and carotenoids [58]. Melanin shields against UV radiation and oxidative stress, also potentially converting radiation into metabolic energy [59]. Some black fungi demonstrate enhanced growth under ionizing radiation, evidencing extraordinary adaptability [60]. Deep-sea fungi from high-pressure, low-temperature, and nutrient-poor sediments contribute to carbon cycling, with species like *Aspergillus terreus* adapting by producing cold-active enzymes [61,62]. These findings underscore fungi’s contributions to biogeochemical cycles even in extreme ecosystems.

Thermotolerance underpins fungal pathogenicity by facilitating adaptation to 37 °C and febrile conditions, enabling fungi such as *C. albicans*, *A. fumigatus*, and *C. neoformans* to colonize and invade hosts [63]. A deeper molecular perspective reveals that the transcription factor Hsf1 is the master regulator responsible for initiating the heat shock response, directly controlling the expression of numerous Heat Shock Proteins (HSPs) like Hsp90. Hsp90, in turn, acts as a molecular chaperone essential for maintaining the stability and function of key fungal kinases and signaling proteins required for morphogenesis (e.g., yeast-to-hyphae switch in *C. albicans*) and virulence at 37 °C. Furthermore, adaptation to high temperatures requires precise remodeling of the cell membrane by adjusting the ratio of saturated to unsaturated fatty acids to maintain membrane fluidity and integrity, a process governed by dedicated desaturase enzymes. This coordinated molecular response, far beyond simple protein folding, is what truly enables survival at mammalian temperatures [64,65].

*A. fumigatus* tolerates temperatures above 50 °C through adaptations including not only heat shock proteins (HSPs), but also thermostable enzymes and membrane lipid remodeling [63,66]. Thermotolerance is a pivotal virulence factor enabling survival at mammalian body temperatures, a prerequisite for many infections [61,67]. Thermotolerance is a pivotal virulence factor enabling survival at mammalian body temperatures, a prerequisite for many infections. The diverse temperature thresholds and underlying mechanisms of clinically relevant fungi are summarized (Table 3).

While thermotolerance is a well-established prerequisite for pathogenicity, significant knowledge gaps persist regarding the signaling hierarchy that links host temperature detection (37 °C/febrile conditions) to the precise regulation of key virulence factors and metabolic adaptation. A key controversy is whether thermotolerance is merely a prerequisite for survival or an actively modulated virulence mechanism, especially in emerging pathogens like *Candiozyma auris* which tolerates >40 °C. This debate informs the clinical relevance of targeting highly conserved stress responses like Hsp90. Future directions should focus on identifying fungus-specific vulnerabilities within the thermal stress response, such as non-conserved components of membrane lipid regulation or the unique metabolic pathways of extremotolerant fungi. Developing adjuvant therapies that target the major regulators of these systems, like Hsf1, may offer a promising strategy to disarm fungal virulence without incurring the systemic toxicity associated with targeting highly conserved proteins.

## 4. Melanin and Stress Resistance

The most studied melanization pathway, the L-3,4-dihydroxyphenylalanine (DOPA) pathway, relies on the critical enzyme laccase (Lac1) in *Cryptococcus neoformans*. Laccase catalyzes the polymerization of DOPA-like precursors derived from catechols found in the environment (e.g., bird droppings) into the mature melanin polymer. Conversely, many filamentous fungi, such as *Aspergillus fumigatus*, utilize the 1,8-dihydroxynaphthalene (DHN) pathway, which involves several reductase and dehydratase enzymes (like the polyketide synthase PksP/Alb1) to produce the pigment. The genetic control of these pathways is a central virulence mechanism, as melanin masks Pathogen-Associated Molecular Patterns (PAMPs), directly interfering with phagocyte recognition and clearance [74,75].

Melanized fungi evade immune clearance by scavenging reactive oxygen species and modulating host responses [76]. Melanin also reduces antifungal efficacy by binding drugs such as amphotericin B [77]. Its dual role in environmental resilience and pathogenesis emphasizes melanin as a key adaptive molecule for fungi inhabiting diverse and hostile niches [78]. Its dual role in environmental resilience and pathogenesis emphasizes melanin as a key adaptive molecule for fungi inhabiting diverse and hostile niches. Examples of melanized fungi, their protective factors, and their adaptive roles are detailed in Table 4.

Although melanin is crucial for both environmental resilience and fungal virulence, significant knowledge gaps persist in understanding the precise mechanisms by which it scavenges reactive oxygen species and modulates host immune responses, particularly the quantitative relationship between melanin density and effective clearance by macrophages. A key controversy lies in the exact mechanism of melanin’s anti-antifungal action, including whether it reduces Amphotericin B efficacy primarily through direct drug binding or by altering cell wall properties that impede drug entry. Future directions should focus on elucidating the melanin biosynthetic pathway as a specific therapeutic target. Research should aim to develop novel compounds that selectively inhibit key melanogenesis enzymes, thereby disarming fungal protection without causing host toxicity.

## 5. Fungal Adaptive Plasticity: Genomic, Epigenetic, and Survival Strategies

Fungal asexual spores (conidia) and sexual spores are highly resistant structures that ensure survival under adverse conditions including extreme temperature, desiccation, UV radiation, and nutrient scarcity. Conidia, ascospores, and basidiospores enable dispersal, dormancy, and infection of hosts. In many medically relevant fungi that lack a known sexual cycle, such as *Aspergillus* species, conidia are the primary infectious propagules and highly resistant survival forms [83]. Molecular mechanisms governing spore formation and persistence are being elucidated, revealing crucial roles in fungal propagation and pathogenicity [84]. Spore melanization further enhances resistance to environmental and host-derived stresses, contributing to long-term survival and disease transmission [85].

Fungal pathogens exhibit extensive genomic and epigenetic plasticity enabling rapid adaptation to environmental and host pressures [86]. Mechanisms include ploidy variations, chromosome rearrangements, copy number variations, and epigenetic changes such as DNA methylation and histone modifications [87,88]. Such plasticity underlies antifungal resistance and virulence modulation, with epigenetic regulation increasingly recognized as a target for novel therapies [89,90].

A hallmark of opportunistic fungal pathogens is their ecological plasticity, allowing them to transition between saprophytic or commensal states and a parasitic lifestyle. Whether originating from the external environment or as a member of the host’s microbiota, this switch to a pathogenic state involves a complex modulation of metabolism, gene expression, and morphology in response to host cues [91]. Successful infection requires the deployment of virulence factors such as hydrolytic enzymes and biofilm formation, enabling tissue invasion and immune evasion [1]. Understanding this duality enhances insights into fungal pathogenesis and potential therapeutic targets.

The rapid adaptability of fungi is frequently controlled by Epigenetic Mechanisms that modify gene expression without altering the underlying DNA sequence. Changes in DNA methylation or histone modifications (acetylation and methylation), mediated by enzymes like histone acetyltransferases (HATs) and deacetylases (HDACs), can quickly silence or activate entire virulence programs, contributing significantly to antifungal resistance and morphological transition [92]. Furthermore, certain cytosolic enzymes exhibit “moonlighting” multifunctions, where they assume non-canonical roles, typically on the cell surface, in response to host cues. For instance, housekeeping proteins involved in glycolysis, such as Enolase or Glyceraldehyde-3-Phosphate Dehydrogenase, are frequently found anchored to the fungal cell wall of *Candida* and *Histoplasma capsulatum* [93]. In this extracellular location, they function as adhesins, plasminogen receptors, or immunomodulators, promoting colonization and tissue invasion. This relocalization, often triggered by stress or host-derived signals, is a highly conserved adaptive strategy for rapid pathogenesis [12].

Recognizing plasticity as central to virulence, significant knowledge gaps persist regarding the specific host signals that acutely trigger genomic and epigenetic rearrangements during infection, and whether this plasticity is a stochastic or rigidly programmed event. A key controversy surrounds the causality between ploidy events and antifungal resistance: is genomic variation a primary defense mechanism or merely a consequence of drug selective pressure? Future directions must utilize single-cell sequencing technologies to map genomic and epigenetic changes in real-time during the commensal-to-pathogen transition, aiming to develop epigenetic drugs that can “lock” the fungus in its saprophytic state, thereby preventing the expression of virulence.

Comprehending this adaptive plasticity offers essential insights into fungal pathogenesis and identifies new points for therapeutic intervention. A comprehensive overview of medically important fungal species, categorized by their ecological lifestyle and associated pathogenic infections, is provided (Table 5).

## 6. Human-Associated Risk Factors and Changes in Fungal Disease Dynamics

The incidence of fungal infections is rising, driven by factors such as population aging, increased use of immunosuppressants, broad-spectrum antibiotics, invasive procedures, and comorbidities including HIV, diabetes, and cancer [118,119]. These conditions disrupt host defenses and create niches favorable to fungal colonization and infection.

Environmental changes, including urbanization, as well as increased international travel and global trade, facilitate the emergence and spread of novel and resistant fungi, complicating diagnosis and treatment [120]. Surveillance and public health measures integrating One Health approaches, acknowledging human, animal, and environmental linkages, are essential [121,122].

Environmental changes, including urbanization, as well as increased international travel and global trade, facilitate the emergence and spread of novel and resistant fungi, complicating diagnosis and treatment. Surveillance and public health measures integrating One Health approaches, acknowledging human, animal, and environmental linkages, are essential. Specific human-associated factors that increase susceptibility to fungal infections are detailed in Table 6.

The emergence of fungal infections is also strongly linked to occupational and environmental exposures, creating specific risk profiles often overlooked in clinical settings. For example, exposure to airborne conidia of *Aspergillus* is significantly higher among construction workers due to disturbed soil and insulation materials [130]. Similarly, individuals engaged in farming, caving, or landscaping face elevated risk of exposure to environmental pathogens like *Histoplasma capsulatum* (found in bird and bat droppings) [131] and *Coccidioides immitis* (found in arid soils) [132]. Recognizing these ecological and occupational links is vital for implementing targeted surveillance and prevention, aligning with the One Health paradigm.

Despite the clear recognition of host-associated risk factors, significant knowledge gaps persist in the precise quantification of each factor’s contribution (e.g., antibiotic use vs. comorbidity) to the individual risk of developing invasive mycosis. A key controversy lies in the practical application of the One Health approach: how to integrate environmental surveillance and sampling (soil, water, animals) into hospital clinical protocols in a cost-effective and scalable manner to prevent outbreaks of emerging and resistant fungi like *Candiozyma auris*? Future directions require the implementation of Machine Learning systems to analyze vast sets of clinical, microbiological, and environmental data. The goal is to develop robust predictive models that can identify high-risk patients before fungal infection and optimize public health interventions that effectively link environmental factors to human health outcomes.

## 7. The Landscape of Antifungal Therapy: From Current Guidelines to Future Innovations

Treating fungal infections remains a significant clinical challenge, compounded by a limited antifungal arsenal and increasing pathogen resistance [133]. The therapeutic landscape is defined by established clinical guidelines for current agents, promising investigational drugs, and revolutionary technologies poised to shape future treatments. Current management of invasive fungal infections relies on three main classes of antifungal drugs, each targeting different fungal cellular mechanisms.

• Polyenes: Amphotericin B is polyene used clinically for invasive mycoses. The drug targets ergosterol, causing the disruption of the fungal cell membrane and compromising its integrity. It is highly effective against a broad range of pathogens, including *Candida* spp. and *Aspergillus* spp., making it a cornerstone for severe systemic infections. Modern lipid formulations have significantly reduced its characteristic nephrotoxicity, allowing for higher doses and improved outcomes [134].
• Azoles: The triazoles, including fluconazole, itraconazole, voriconazole, and others, function by inhibiting the enzyme lanosterol 14α-demethylase, which blocks ergosterol biosynthesis and disrupts membrane integrity [135]. Fluconazole, for example, is widely used for candidiasis due to its favorable safety profile, but it has limited efficacy against intrinsically resistant species like *N. glabrata* and *Candida krusei* [28,100].
• Echinocandins: This class, which includes caspofungin, micafungin, and anidulafungin, offers a high degree of selectivity by inhibiting β-(1,3)-d-glucan synthesis, a critical component for the synthesis of the fungal cell wall, a machinery that is absent in animal cells [136]. Due to their proven efficacy and safety, echinocandins are recommended as first-line therapy for invasive fungal infections such as candidemia [137]. When they are unavailable or contraindicated, alternatives like a triazole or liposomal amphotericin B are selected based on infection severity and species susceptibility [137,138]. According to current guidelines, the treatment duration for candidemia typically extends for 14 days after the first negative blood culture, necessitating serial monitoring to confirm the clearance of the pathogen [139].

In parallel with established protocols, innovative modalities are under intense investigation to address the rise of antifungal resistance. Recently available and forthcoming drugs are targeting novel fungal processes to overcome the limitations of conventional agents. Promising new antifungals, such as the once-weekly echinocandin rezafungin, the novel triterpenoid ibrexafungerp, and the first-in-class agent fosmanogepix, show broad efficacy against resistant yeasts and molds [133,140]. Beyond new molecules, adjunctive strategies are also being explored. Photodynamic therapy (PDT), which uses photosensitizers activated by light to generate reactive oxygen species, has shown promise in disrupting biofilms and enhancing the susceptibility of resistant *Candida* strains to traditional antifungals like amphotericin B [141].

The increasing burden of fungal disease has catalyzed a wave of innovation, leveraging cutting-edge technologies to advance diagnostics and drug development.

• Artificial intelligence (AI): Modern antifungal research is driven by a synergy between large-scale data analysis and precise genetic engineering. Advances in genomic, transcriptomic, and proteomic analyses generate massive datasets that elucidate pathogen virulence and resistance pathways [142]. AI then harnesses this information, using machine learning to rapidly identify novel compounds, predict resistance mutations, and optimize therapeutic regimens [143]. Complementing these computational strategies, gene-editing tools like CRISPR/Cas provide the means for functional validation, allowing researchers to precisely dissect fungal pathogenicity and confirm novel therapeutic targets in preclinical models [144,145]. Together, these technologies are creating a powerful pipeline for developing personalized antifungal therapies.
• Nanotechnology and Biomaterials: Nanoparticles, hydrogels, and polymer-based materials are being developed for targeted drug delivery and the prevention of biofilm formation on medical devices [146]. Materials like silver nanoparticles can disrupt fungal membranes, representing a promising adjunctive therapy [147]. Similarly, novel platforms such as biodegradable, nitric oxide (NO)-releasing microparticles have emerged as a powerful strategy, proving effective against a wide range of pathogenic fungi and their biofilms [148].
• Immunotherapy and Vaccines: Emerging immunotherapies, including monoclonal antibodies and fungal vaccines, are under development to enhance host immunity. These approaches aim to prevent infection or serve as adjuncts to traditional antifungals, offering a powerful new strategy in the fight against fungal pathogens [149].
• Diagnostic Innovations: Advances in rapid molecular diagnostics, combined with machine learning analysis of clinical and imaging data, are set to improve the timely detection and personalized treatment of fungal infections [150,151].

Finally, the effective and sustainable use of this entire therapeutic arsenal, both current and future, depends on robust control strategies. Antifungal stewardship programs, often managed by clinical pharmacists and infectious diseases physicians, are essential to curb the development of resistance and optimize patient outcomes. These programs, combined with epidemiological surveillance and continuous education for healthcare providers, are critical components for managing fungal infections effectively in both hospital and community settings [152].

Despite the promise of new agent classes (like rezafungin and ibrexafungerp) and emerging technologies (AI, nanotechnology), a critical knowledge gap is the lack of understanding of fungal compensatory mechanisms activated when primary target pathways are blocked, which explains the rapid emergence of cross-resistance. A central controversy lies in the clinical efficacy of emerging adjunctive therapies (such as PDT and immunotherapy), which still lack robust, large-scale clinical trials to determine their role as the standard of care compared to conventional monotherapy. Future directions must focus on optimizing strategies that attack multiple pathways simultaneously—using AI to design drug combinations that overcome metabolic redundancy, nanotechnology to ensure the targeted and non-toxic delivery of these cocktails, and immunotherapies (vaccines) that provide long-term protection against common opportunistic pathogens like *C. albicans* and *A. fumigatus*.

## 8. Advances in Diagnosis of Fungal Infections

Accurate and timely diagnosis of fungal infections remains a critical challenge in clinical mycology. Conventional methods such as direct microscopy, histopathology, and culture remain gold standards but are limited by low sensitivity, slow turnaround times, and difficulty in species-level identification [153].

Recent advances include molecular diagnostic assays such as PCR-based tests and proteomic technologies like MALDI-TOF mass spectrometry that enable rapid and precise species identification within hours instead of days or weeks [154]. MALDI-TOF has revolutionized fungal diagnostics by accurately identifying over 50 fungal species from clinical specimens, dramatically reducing the diagnostic window and allowing earlier targeted antifungal therapy [155].

Non-invasive biomarkers such as 1,3-β-d-glucan and galactomannan assays provide useful adjuncts for invasive fungal infection detection, especially in high-risk patients. While these biomarkers exhibit high negative predictive values, their specificity and positive predictive values remain limited, necessitating integration with clinical and radiological findings [156]. Current guidelines recommend combining biomarker assays with molecular diagnostics for optimal management. Integration of advanced imaging modalities (e.g., CT, MRI) with AI-driven image analysis algorithms offers promising tools for early detection and differentiation of fungal infections, facilitating personalized care [157].

Despite these advances, limitations in accessibility, cost, and standardization constrain widespread implementation, particularly in low-resource settings. Research efforts are ongoing to develop point-of-care diagnostics, multiplex assays, and digital health solutions to address these gaps and improve global fungal infection management [158].

Despite the speed of MALDI-TOF and the utility of biomarkers, a critical knowledge gap in fungal diagnosis is the validity and sensitivity of molecular assays (PCR) directly in blood specimens for non-candidemic fungemias and deep invasive fungal infections, often limited by low fungal loads and PCR inhibitors. A key controversy lies in the interpretation and standardization of pan-fungal biomarkers, whose positive results can be influenced by non-infection-related clinical procedures or drugs. Future directions must focus on developing low-cost, high-sensitivity point-of-care (POC) tests that combine molecular detection with multiplex biomarker analysis, leveraging AI to integrate imaging and clinical findings. This is crucial for overcoming accessibility constraints and enabling timely detection and personalized therapies globally.

## 9. Future Prophylaxis: Immunotherapy and Vaccine Development

Preventing fungal infections is a crucial public health priority, considering the increasing incidence and limited therapeutic options [159]. Effective prevention strategies target both environmental and host-related factors. Maintaining personal hygiene, reducing exposure to contaminated surfaces, and avoiding sharing of personal items are fundamental. For example, avoiding walking barefoot in communal areas, keeping skin dry, and using breathable clothing help prevent dermatophytosis and candidiasis [160,161]. Proper cleaning and disinfection in healthcare settings are essential to limit nosocomial fungal transmission, particularly of opportunistic pathogens like *Candida* and *Aspergillus* spp. [160,161]. Minimizing fungal spore exposure in high-risk environments via air filtration, humidity control, and protective equipment reduces the risk of invasive infections, especially in immunocompromised patients [162].

Beyond direct-acting antifungal agents, a significant frontier is the modulation of the host’s own immune system. Although no licensed fungal vaccines currently exist, research into candidates targeting major pathogens is advancing, offering hope for future prophylaxis in vulnerable populations [163]. In parallel, immunotherapies designed to augment host defenses, such as monoclonal antibodies, checkpoint inhibitors, and engineered immune cells, are under active development to improve the host’s ability to resist and clear invasive fungal infections. Public health strategies for fungal diseases are increasingly aligned with major global health paradigms. For example, modeled on the UNAIDS 95-95-95 targets for HIV, the World Health Organization (WHO) advocates for a ‘95-95’ approach to manage cryptococcal meningitis, a leading cause of death in people with advanced HIV disease. This initiative aims to ensure 95% of eligible patients are screened for the cryptococcal antigen and 95% of positive cases receive effective antifungal therapy [164].

Despite the importance of prevention, a major knowledge gap lies in the identification of optimal antigenic targets for the development of fungal vaccines. The poor understanding of protective immunity mechanisms against fungi (host–fungus immunology) hinders the rational design of universal candidates. A key controversy is the efficacy and safety of prophylactic and preemptive antifungal use in low-to-moderate-risk patients; generalization may increase selective pressure and resistance without clear benefit. Future directions must focus on advanced phase trials of therapeutic vaccines and specific immunological adjuvants, such as monoclonal antibodies targeting virulence molecules or surface antigens. Furthermore, the application of goal-based prevention programs (like the WHO’s ‘95-95’ initiative) needs to be expanded and adapted to other endemic and opportunistic mycoses to become a global paradigm.

## 10. Challenges and Future Perspectives in Fungal Infection Research and Treatment

Fungal infections pose a growing global health threat, a challenge formally recognized by the World Health Organization’s (WHO) inaugural Fungal Priority Pathogens List. This threat is further complicated by rising antifungal resistance, climate change, and evolving host and environmental factors [165,166]. Despite advances in diagnostics and therapeutics, significant barriers remain, including limited drug classes, diagnostic delays, and insufficient understanding of complex host–pathogen interactions [167].

Recent approvals of new antifungal agents targeting unique fungal pathways provide hope for overcoming multidrug resistance. Research into natural products, drug repurpose, immunotherapies, and nanotechnology-based formulations are ongoing to expand our therapeutic arsenal [145,168]. Emerging molecular diagnostics and biomarker assays offer faster, more accurate detection, enabling earlier interventions [169]. Meanwhile, combined antifungal therapies and stewardship programs improve treatment outcomes and mitigate resistance emergence [170]. Effective management of fungal diseases requires integrated “One Health” efforts across human health, veterinary medicine, and environmental science, recognizing the interconnectedness of ecosystems influencing fungal epidemiology [171].

Despite the global recognition of the fungal threat by the WHO Priority Pathogens List, a major knowledge gap lies in the mechanistic understanding of the triadic interaction (fungus-host-environment). It remains unclear how climate change and pollution predictably modulate fungal virulence and drug resistance. A central controversy is the economic and logistical feasibility of implementing the One Health concept on a large scale, as environmental surveillance and high-resolution genomic sequencing are not yet routine or accessible in low-resource clinical settings. Future directions must focus on creating global surveillance networks that integrate genomic, environmental, and clinical data, leveraging Artificial Intelligence (AI) to predict outbreaks and optimize antifungal stewardship strategies. The emphasis should shift towards developing a universal pan-fungal vaccine or utilizing precision therapies guided by host-response biomarkers to modulate patient immunity, rather than relying solely on new antifungal agents.

## 11. Conclusions

Fungal pathogens demonstrate extraordinary versatility through an array of biochemical, morphological, and genetic adaptations that enable survival across diverse environments and host niches. Hydrolytic enzymes play pivotal roles in nutrient acquisition, tissue invasion, and immune evasion, while metabolic flexibility and stress tolerance augment pathogenic potential. The emergence of antifungal resistance, driven by genetic mutations, epigenetic shifts, and biofilm-associated tolerance, complicates treatment and necessitates innovative therapeutic strategies. The time for incremental advancements in clinical mycology has passed. Fungal pathogens represent a quintessential One Health threat, leveraging complex genomic and epigenetic plasticity to bridge the gap “From the Ground to the Clinic.” The central challenge is recognizing that fungal virulence is not static but a dynamic, environment-driven phenomenon mediated by specific molecular switches—from the Lac1-driven melanization to Hsf1-regulated thermotolerance. Understanding these complex interactions between host, pathogen, and environment is essential to enhance diagnosis, prevention, and management of fungal diseases. To curtail the escalating global crisis of antifungal resistance, we must move beyond the current limited therapeutic arsenal. Future efforts must be bold: harnessing AI and single-cell omics to decode the host–pathogen-environment triad, designing precision epigenetic drugs to “lock” the fungus in its non-pathogenic state, and prioritizing the long-sought goal of an effective pan-fungal vaccine. Only by integrating molecular authority with a global surveillance strategy can we hope to contain this adaptable and ever-evolving threat.

## Figures and Tables

**Table 1:** Examples of competitive strategies for resource acquisition in fungi.

Fungus	Primary Carbon Sources	Carbon Metabolism Strategy	Environmental Adaptability	Reference
*Aspergillus fumigatus*	Amino acids, organic acids, simple sugars	Versatile use of fermentable and non-fermentable compounds; Urea/Nitrogen metabolism	Thrives in decaying organic matter and human lungs	[23]
*Candida albicans*	Glucose, lipids, host-derived sugars (e.g., galactose)	Versatile metabolism of diverse sugars/amino acids; Fermentation and respiration linked to oxygen	Adapts to mucosal surfaces and systemic infections	[24]
*Penicillium* spp.	Cellulose, lignin	Ammonium, nitrate, amino acids	Decomposes plant material in soil and organic matter	[25]
*Cryptococcus neoformans*	Glucose, lactate, fatty acids	Urea and diverse nitrogen metabolism; Capsule synthesis linked to N availability	Survives in the brain during cryptococcal meningitis	[26]
*Fusarium solani*	Polysaccharides, organic acids	Amino acids, peptides	Adapts to nitrogen-poor soil and plant infections	[27]
*Pichia kudriavzevii*	Acetate, glycerol	Acidic environment adaptation	Colonizes mucosal surfaces, resists antifungals	[28]
*Trichoderma* spp.	Lignocellulosic material	Various organic and inorganic nitrogen sources	Dominates soil microbiomes, biological control agent	[29]
*Mucor circinelloides*	Sugars, organic acids, alcohols	Fermentation and respiration based on oxygen	Saprophytic growth in decomposing organic matter	[30]
*Aspergillus niger*	Starch, cellulose, pectin	Diverse nitrogen assimilation	Outcompetes microbes in nutrient-rich environments	[31]
*Basidiobolus ranarum*	Lipids, proteins, carbohydrates	Adapted to varied nitrogen sources	Found in soil, decaying matter, amphibian hosts	[32]
*Exophiala dermatitidis*	Hydrocarbons, phenolic compounds, simple sugars	Flexible nitrogen metabolism	Survives in extreme environments (hot springs, pollution)	[33]
*Sporothrix schenckii*	Host-derived amino acids, lipids	Melanin production for protection	Adapts to host tissues, causing sporotrichosis	[34]
*Rhizopus oryzae*	Starches, lipids, proteins	Rapid colonization of decaying material	Grows on spoiled food and agricultural waste	[35]

**Table 2:** Examples of fungal strategies for organic matter decomposition, tissue invasion, and immune evasion.

Fungus	Organic Material Breakdown	Human Tissue Invasion	Hydrolytic Enzyme Production & Regulation	Biofilm Formation	Host Defense Evasion	Reference
*Aspergillus niger*	Produces proteases, amylases, and cellulases to decompose organic matter	Produces collagenases for tissue invasion	Regulated by nutrient availability and environmental cues	Forms biofilms on decaying matter and in tissues	Proteases evade immune response, promoting infection	[43]
*Trichophyton species*	Degrades keratin and produces amylase for substrate breakdown	Keratinase production for skin and nail infections	Gene expression activated during skin invasion	Forms biofilms on skin and nails	Keratinases allow penetration into skin and nails	[44]
*Candida albicans*	Degrades lipids and proteins for biofilm and tissue invasion	Phospholipases aid mucosal invasion, SAPs for biofilm formation	Enzyme production triggered by host signals	Known for biofilm formation on medical devices	SAPs degrade immune proteins, evading immune response	[24]
*Fusarium species*	Breaks down plant materials with cellulases and amylases	Collagenases invade deeper tissues in immunocompromised hosts	Dual regulation: by host immune system & environmental cues (pH, nutrients).	Forms biofilms in tissues, contributing to chronic infections	Collagenases assist in evading immune defenses	[45]
*Aspergillus fumigatus*	Breaks down organic matter with proteases and lipases	Elastases and collagenases invade lung and vascular tissue	Regulated by inflammation and immune responses	Biofilm formation in lung tissue and other surfaces	Proteases degrade immune molecules, evading phagocytosis	[23]
*Histoplasma capsulatum*	Produces proteases and lipases for tissue invasion	Invades lung tissue using proteases and lipases	Regulated by host immune signals during infection	Biofilm formation in host tissues	Degrades immune molecules to evade immunity	[46]
*Coccidioides immitis*	Produces proteases for organic material breakdown	Collagenase production for lung tissue invasion	Regulated by host environmental cues	Forms biofilms in the lung and other tissues	Protease production aids immune evasion	[47]
*Sporothrix schenckii*	Produces elastases and proteases for tissue invasion	Uses elastases and collagenases to invade connective tissue	Regulated by immune responses and environmental stress	Forms biofilms on skin and connective tissue; bioinformatic predictions suggest potential for biofilm formation, though experimental validation is necessary	Elastases help the fungus penetrate host tissues	[34]

**Table 3:** Examples of thermotolerance mechanisms in medically relevant fungi.

Fungus	Maximum Growth Temperature	Key Thermotolerance Mechanisms	Reference
*Aspergillus fumigatus*	~50 °C	- Membrane lipid stability - Heat-responsive gene expression	[68]
*Blastomyces dermatitidis*	~39 °C	- Dimorphic switching (mold to yeast) - Upregulation of HSPs and stress-response genes	[69]
*Candida albicans*	~37 °C	- Heat shock protein (HSP) production - Morphological switching (yeast to hyphae)	[24]
*Candiozyma auris*	~42 °C	- Membrane modifications to withstand heat - High resilience against environmental and thermal stresses	[70]
*Coccidioides immitis*	~42 °C	- Transition to spherule form at human body temperature - Stress - Response proteins to manage thermal stress	[47]
*Cryptococcus neoformans*	~40 °C	- Melanin production for cellular stability under heat	[71]
*Histoplasma capsulatum*	~37 °C	- Thermal dimorphism (yeast form at 37 °C)	[69]
*Paracoccidioides brasiliensis*	~37 °C	- Dimorphic behavior (mold to yeast) - HSPs and oxidative stress management	[72]
*Sporothrix schenckii*	~37 °C	- Adaptations for growth at elevated temperatures - Altered cell wall composition for thermal stress tolerance	[34]
*Talaromyces marneffei*	~37 °C	- Thermal dimorphism - Regulation of key genes during temperature transitions	[73]

**Table 4:** Examples of melanized fungi: UV protection, oxidative stress resistance, and pathogenic factors.

Fungus	UV Protection	Immune Response Modulation	Environmental Adaptation	Reference
*Cryptococcus neoformans*	Protects against UV radiation in bird droppings	Inhibits phagocytosis	Survives in diverse niches, including the human body	[79]
*Exophilia dermatitidis*	Resists UV radiation in aquatic environments	Inhibits phagocytosis	Survives in aquatic and soil environments	[60]
*Alternaria alternata*	Protects against UV, surviving in outdoor environments	Modulates host immune response to promote fungal persistence.	Survives in various environments, including plants	[80]
*Exophiala dermatitidis*	Survives in outdoor environments with UV radiation	Modulates immune response via melanin and intracellular survival	Adaptable to aquatic and terrestrial environments	[33]
*Madurella mycetomatis*	UV resistance in soil environments	It scavenges ROS from immune cells and masks cell wall β-glucans to prevent immune recognition.	Survives in extreme environmental conditions	[81]
*Fonsecaea pedrosoi*	Protects against UV in soil and human skin	Forms phagocytosis-resistant muriform cells; its melanin layer also scavenges ROS and masks PAMPs	UV resistant, adapts to soil and human skin	[82]

**Table 5:** Examples of medically important fungal species, their lifestyles, and associated infections.

Species	Lifestyle	Pathogenic Infection	Reference
*Acremonium falciforme*	Saprophytic (soil, decaying organic matter)	Cutaneous, corneal infections	[94]
*Alternaria alternata*	Saprophytic (decaying vegetation)	Pulmonary infections, allergic reactions	[95]
*Aspergillus fumigatus*	Saprophytic (decaying vegetation)	Pulmonary infection, invasive aspergillosis	[68]
*Aspergillus flavus*	Saprophytic (soil, decaying matter)	Aspergillosis, keratitis, otomycosis	[96]
*Aspergillus niger*	Saprophytic (decaying vegetation)	Otomycosis, pulmonary aspergillosis	[31]
*Blastomyces dermatitidis*	Saprophytic (decaying wood, soil)	Pulmonary and disseminated infection	[69]
*Bipolaris* spp.	Saprophytic (plants)	Respiratory and cutaneous infections	[97]
*Candida albicans*	Normal microbiota (skin, mouth, gastrointestinal tract)	Oral, vaginal and systemic	[98]
*Candidozyma auris*	Opportunistic pathogen; colonizes skin, serving as a reservoir for healthcare-associated infections	Skin colonization and disseminated infection	[99]
*Nakaseomyces glabrata*	Normal microbiota (gastrointestinal tract)	Invasive candidiasis	[100]
*Candida tropicalis*	Normal microbiota	Invasive candidiasis	[100]
*Cladosporium cladosporioides*	Saprophytic (decaying organic matter)	Respiratory infections, allergies	[101]
*Coccidioides immitis*	Saprophytic (arid soil)	Pulmonary and disseminated infection	[69]
*Cryptococcus neoformans*	Saprophytic (soil, excrement)	Meningoencephalitis, pneumonia, CNS infection	[98]
*Cryptococcus gattii*	Saprophytic (trees, soil)	Meningoencephalitis, pneumonia, CNS infection	[102]
*Exophiala dermatitidis*	Saprophytic (soil, organic matter)	Cutaneous, subcutaneous infections	[33]
*Fusarium* spp.	Saprophytic (soil, decaying plants)	Keratitis, onychomycosis, disseminated infection	[103]
*Fusarium verticillioides*	Saprophytic (decaying plants)	Pulmonary and systemic infections	[104]
*Histoplasma capsulatum*	Saprophytic (soil contaminated with bird and bat droppings)	Pulmonary infection, disseminated infection	[69]
*Lomentospora prolificans*	Saprophytic (soil, organic matter)	Invasive infections (post-transplant, leukemia)	[105]
*Microsporum canis*	Zoophilic	Dermatophytosis (tinea capitis, tinea corporis)	[106]
*Nannizzia gypsea* (ex-*Microsporum gypseum*)	Geophilic saprophyte; lives on keratin in soil.	Dermatophytosis (tinea corporis, tinea capitis)	[107]
*Paracoccidioides brasiliensis*	Saprophytic (tropical soil, organic matter)	Pulmonary and disseminated infection	[72]
*Paracoccidioides lutzii*	Saprophytic (soil)	Pulmonary and disseminated infection	[72]
*Talaromyces marneffei*	Saprophytic (soil, organic matter)	Systemic infections (HIV/AIDS patients)	[108]
*Pneumocystis jirovecii*	Opportunistic pathogen; asymptomatically colonizes the respiratory tract of immunocompetent hosts.	Pneumonia in immunocompromised patients	[109]
*Rhizopus* spp. (Mucormycetes)	Saprophytic (decaying organic matter)	Skin, sinuses and lung infections	[110]
*Scedosporium apiospermum*	Saprophytic (soil, water)	Pulmonary and CNS infection	[111]
*Sporothrix schenckii*	Saprophytic (soil, wood)	Skin and lymphocutaneous infection	[112]
*Sporothrix brasiliensis*	Saprophytic (soil, wood) and Zoonotic	Skin and lymphocutaneous infection	[112]
*Trichophyton mentagrophytes*	Zoophilic	Dermatophytosis (tinea corporis, tinea capitis)	[113]
*Trichosporon asahii*	Normal microbiota (skin, gastrointestinal tract)	Invasive trichosporonosis, bloodstream and urinary tract infections	[114]
*Trichosporon inkin*	Normal microbiota (skin)	White piedra, superficial infections, meningitis	[115]
*Trichosporon mucoides*	Normal microbiota (skin, mucosa)	Skin/hair, bloodstream infection	[116]
*Ustilago maydis*	Saprophytic (organic matter)	Rarely pathogenic to humans; phytopathogen	[117]

**Table 6:** Examples of human risk factors and their impact on fungal infections.

Risk Factor	Description	Fungal Infections Associated	Reference
Immunosuppression	Weakened immune system due to HIV, cancer treatments, etc.	*Cryptococcus neoformans*, *Candida albicans*, *Aspergillus fumigatus*	[16]
Use of Medical Devices	Devices that breach body barriers (e.g., catheters, implants)	*Candida* (candidemia), *Aspergillus*, *Mucor*	[123]
Broad-Spectrum Antibiotics	Disruption of normal microbiota leading to fungal overgrowth	*Candida* (oral thrush, vaginal candidiasis), *Aspergillus*, *Fusarium*	[124]
Chronic Diseases	Conditions like diabetes, liver/kidney disease	*Candida* (candidiasis), *Mucor* (mucormycosis)	[125]
Age	Vulnerabilities due to immature or declining immune systems	*Candida*, *Aspergillus*	[126]
Nutritional Deficiencies	Poor nutrition weakening immune defenses	Invasive fungal infections like *Aspergillus*	[127]
Hospital Settings	High exposure to fungi in intensive care units	*Aspergillus*, *Candida*, *Mucor*	[128]
Smoking & Chronic Lung Diseases	Respiratory damage (increased fungal infection risk)	*Aspergillus*	[129]

## Data Availability

No new data were created or analyzed in this study. Data sharing is not applicable to this article.

## Abbreviations

The following abbreviations are used in this manuscript:

AI	Artificial Intelligence
AI-driven	Artificial Intelligence-driven
CNS	Central Nervous System
CRISPR/Cas	Clustered Regularly Interspaced Short Palindromic Repeats/CRISPR-associated system
CT	Computed Tomography
DNA	Deoxyribonucleic Acid
HIV/AIDS	Human Immunodeficiency Virus/Acquired Immunodeficiency Syndrome
HSPs	Heat Shock Proteins
MALDI-TOF	Matrix-Assisted Laser Desorption/Ionization Time-of-Flight
MRI	Magnetic Resonance Imaging
NO	Nitric Oxide
PAMPs	Pathogen-Associated Molecular Patterns
PCR	Polymerase Chain Reaction
PDT	Photodynamic Therapy
ROS	Reactive Oxygen Species
SAPSs	Secreted Aspartic Proteinases
UNAIDS	Joint United Nations Programme on HIV/AIDS
UV	Ultraviolet
WHO	World Health Organization
